# Dichotomous frequency-dependent phase synchrony in the sensorimotor network characterizes simplistic movement

**DOI:** 10.1038/s41598-024-62848-9

**Published:** 2024-05-24

**Authors:** Vivek P. Buch, Cameron Brandon, Ashwin G. Ramayya, Timothy H. Lucas, Andrew G. Richardson

**Affiliations:** 1 Department of Neurosurgery, School of Medicine, Stanford University, Palo Alto, CA 94304 USA; 2 Department of Neurosurgery, Perelman School of Medicine, University of Pennsylvania, Philadelphia, PA 19104 USA; 3 Departments of Neurosurgery and Biomedical Engineering, The Ohio State University, Columbus, OH 43210 USA

**Keywords:** Network models, Neural decoding

## Abstract

It is hypothesized that disparate brain regions interact via synchronous activity to control behavior. The nature of these interconnected ensembles remains an area of active investigation, and particularly the role of high frequency synchronous activity in simplistic behavior is not well known. Using intracranial electroencephalography, we explored the spectral dynamics and network connectivity of sensorimotor cortical activity during a simple motor task in seven epilepsy patients. Confirming prior work, we see a “spectral tilt” (increased high-frequency (HF, 70–100 Hz) and decreased low-frequency (LF, 3–33 Hz) broadband oscillatory activity) in motor regions during movement compared to rest, as well as an increase in LF synchrony between these regions using time-resolved phase-locking. We then explored this phenomenon in high frequency and found a robust but opposite effect, where time-resolved HF broadband phase-locking significantly decreased during movement. This “connectivity tilt” (increased LF synchrony and decreased HF synchrony) displayed a graded anatomical dependency, with the most robust pattern occurring in primary sensorimotor cortical interactions and less robust pattern occurring in associative cortical interactions. Connectivity in theta (3–7 Hz) and high beta (23–27 Hz) range had the most prominent low frequency contribution during movement, with theta synchrony building gradually while high beta having the most prominent effect immediately following the cue. There was a relatively sharp, opposite transition point in both the spectral and connectivity tilt at approximately 35 Hz. These findings support the hypothesis that task-relevant high-frequency spectral activity is stochastic and that the decrease in high-frequency synchrony may facilitate enhanced low frequency phase coupling and interregional communication. Thus, the “connectivity tilt” may characterize behaviorally meaningful cortical interactions.

## Introduction

Communication between disparate brain regions as measured by network oscillatory activity has long been considered an important feature of many brain functions, including the preparation and execution of movement^1,2^. Network oscillations modify the likelihood and timing of neuronal spiking^3^, leading to spike timing dependent plasticity and synaptic potentiation^4–6^, as well as regulate flow of information by adjusting the network gain during neuronal firing^7^. In neuronal populations involved with movement, a “spectral tilt” phenomenon has been described where broadband oscillatory activity in high frequencies (> 75 Hz) increase, while broadband activity in low frequencies (< 33 Hz) decrease^8^.

Connectivity mediated by network oscillations can be measured both structurally and functionally based on neuroimaging or neurophysiology modalities^9–11^. Functional connectivity assesses the statistical dependence of two neural signals, and can be measured by metrics such as time-series correlation, spectral coherence, or phase synchrony^12–14^. Noninvasive neurophysiology signals have shown sensorimotor cortex task-related increases in coherence^15,16^ and phase synchrony^17,18^ implicating inter-regional functional coupling with movement.

Focal electrocorticography (ECoG)-based analysis of connectivity during motor tasks has been used to improve motor brain-computer interface devices^19,20^. However, intracranial ECoG in more broadly placed frontoparietal regions have been less utilized to explore functional connectivity patterns of movement in the sensorimotor network. Using this intracranial surface recording technique, we previously found that increased low frequency (3–8 Hz, theta band) connectivity was related to increased high frequency spectral power during movement^21^. But changes in high frequency (70–100 Hz, gamma band) connectivity, which has previously been shown to be important for cerebellar cortex information processing during movements^22^, were not examined. Here, we re-examined these intracranial ECoG data to test the hypothesis that low frequency as well as high frequency connectivity, in the form of phase synchrony, provide synergistic but distinct information in sensorimotor network nodes during movement. Specifically, we analyzed intracranial cortical signals from frontoparietotemporal regions in epilepsy patients undergoing clinical monitoring. We assessed changes in stimulus-evoked functional connectivity in both low (3–33 Hz) and high frequency (70–100 Hz) broadbands, similar to the broadbands used to discover the “spectral tilt” oscillatory phenomenon^8^, between various cortical nodes using phase-locking values (PLV)^13^. We then further resolved the analysis into non-overlapping narrowband frequency bins of 4 Hz increments, centered from 5 to 100 Hz, to discern more continuously sampled connectivity patterns associated with movement. We then mapped the time-resolved movement-induced connectivity profile of each pairwise interaction to their anatomical correlate. Finally, we compared these findings to a series of control nodes in the limbic network to identify functional connectivity profiles that may define network activation and regional integration states during movement.

## Results

**Figure 1:**
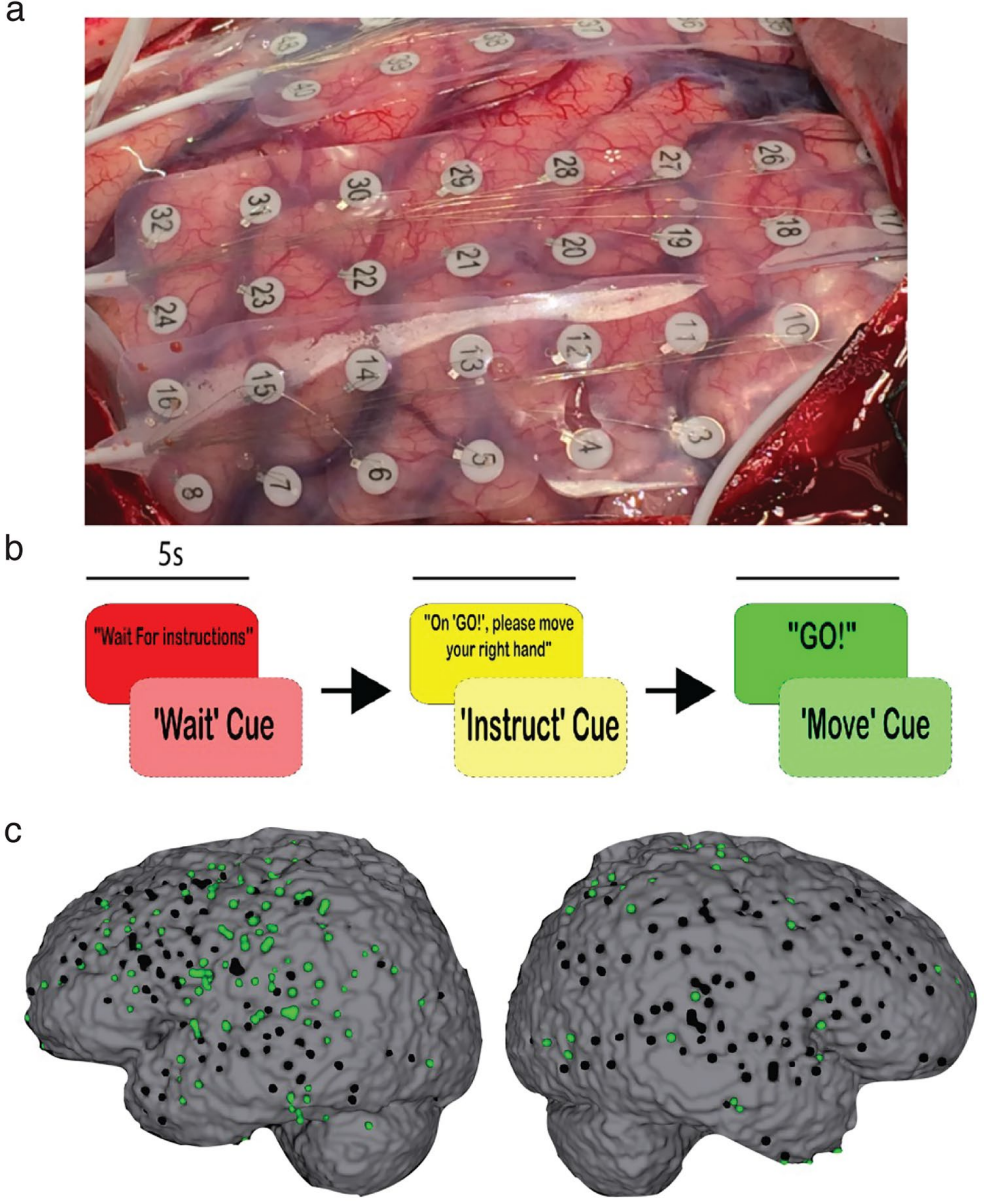
Motor task and electrode localization. (**a**) Single subject example grid placement. (**b**) Task paradigm consisted of three 5 s epochs. The first epoch was the cued “wait” period where subjects were awaiting instruction on the movement to make. The second epoch was the cued instruction period, which tells subjects what to move after the go cue. The third epoch was the go cue, or “move” epoch. (**c**) Distribution of task modulated (green) versus unmodulated (black) intracranial sites for all subjects based on significant high frequency activity changes during the move epoch relative to the wait epoch.

We recorded from intracranial electrodes in seven epilepsy patients (Fig. 1a). During the neural recordings, the subjects performed a simple motor task with trials composed of wait, instruct, and move cues after which the subject performed the instructed movement (Fig. 1b). Similar to prior work, we first identified task-modulated recording sites to characterize the spectral profile of these activated regions^23^. Spectral power in the high gamma band (70–200 Hz), which we refer to as high frequency activity (HFA), was calculated during move and wait periods. Task-modulated sites were defined as those in which the mean difference in HFA between move and wait periods was significantly different from zero, using a two-tailed t-test with a false-discovery rate (FDR) corrected significance threshold of *p* = 0.05^23^. This resulted in 194 task-modulated sites across 5 subjects (Fig. 1c). Two subjects had no sites that met the criterion and thus were excluded from subsequent analysis.

**Figure 2:**
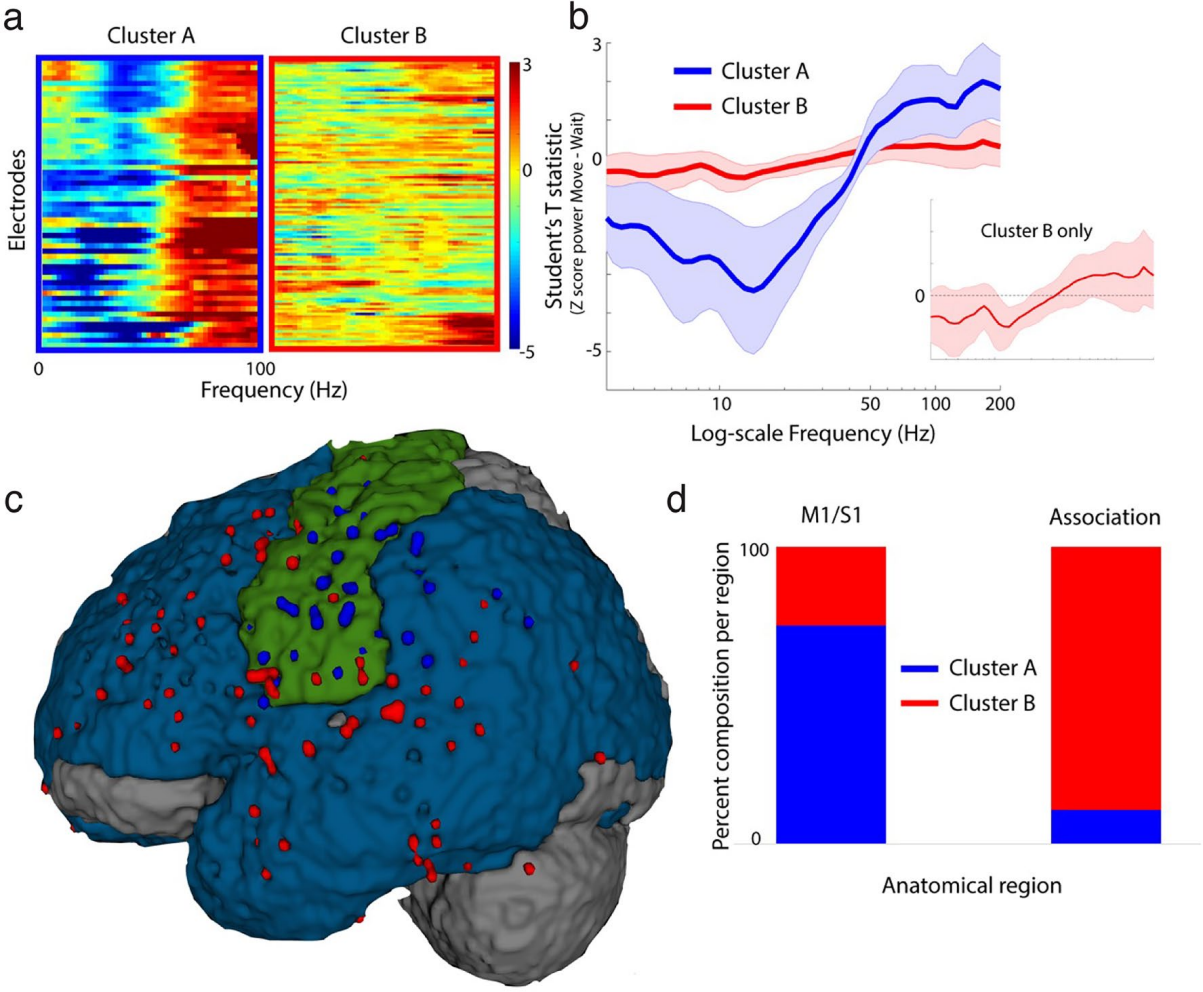
Anatomical distribution of spectral power changes with movement. (**a**) K-means clustering of power spectra change (move epoch relative to wait epoch) across all task-modulated sites in all subjects. (**b**) Mean (± st.dev) spectral power change due to movement for each cluster. (**c**) Anatomical distribution of Cluster A (blue dots) versus Cluster B (red dots) sites. Standardized brain primary sensorimotor cortex (M1/S1) shown in green. Associative (non-primary) frontal, temporal, and parietal lobe shown in cyan. (**d**) Proportion of Clusters A and B in primary motor and sensory cortex (M1/S1) versus association regions.

Next, we quantified patterns of oscillatory activity for all task-modulated sites using z-transformed time–frequency power estimates. For each trial, the spectrograms were aligned to the move and wait cues, subtracted, and averaged over time. Then, across trials, the student’s t statistic was calculated for each frequency bin. We observed that, across the population of modulated sites, the spectral profiles separated into two distinct groups using k-means clustering^24^ and the Calinski-Harabasz criterion^25^ (Fig. 2a). We found that Cluster A was characterized by significantly increased HFA and decreased low frequency activity (LFA, 3–33 Hz) during movement. Cluster B was characterized by a less robust but similar profile (Fig. 2b).

Next, we mapped the two clusters to their anatomical locations (Fig. 2c) and observed a dichotomous relationship. Cluster A was comprised of predominantly primary motor (M1) or primary sensory (S1) cortical sites (n = 47/62, 76%) with a small contribution only from immediately surrounding association regions (n = 15/62, 24%). Cluster B was comprised largely of disparate sites in frontal, temporal, and parietal association regions (n = 115/132, 87%) with minimal contribution from primary sensorimotor cortex (n = 17/132, 13%) (Fig. 2d). Thus, confirming prior studies, a “spectral tilt” was observed in task-modulated sites, most robustly in the primary sensorimotor region, where movement caused a decrease in LFA and an increase in HFA relative to rest^8^.

**Figure 3:**
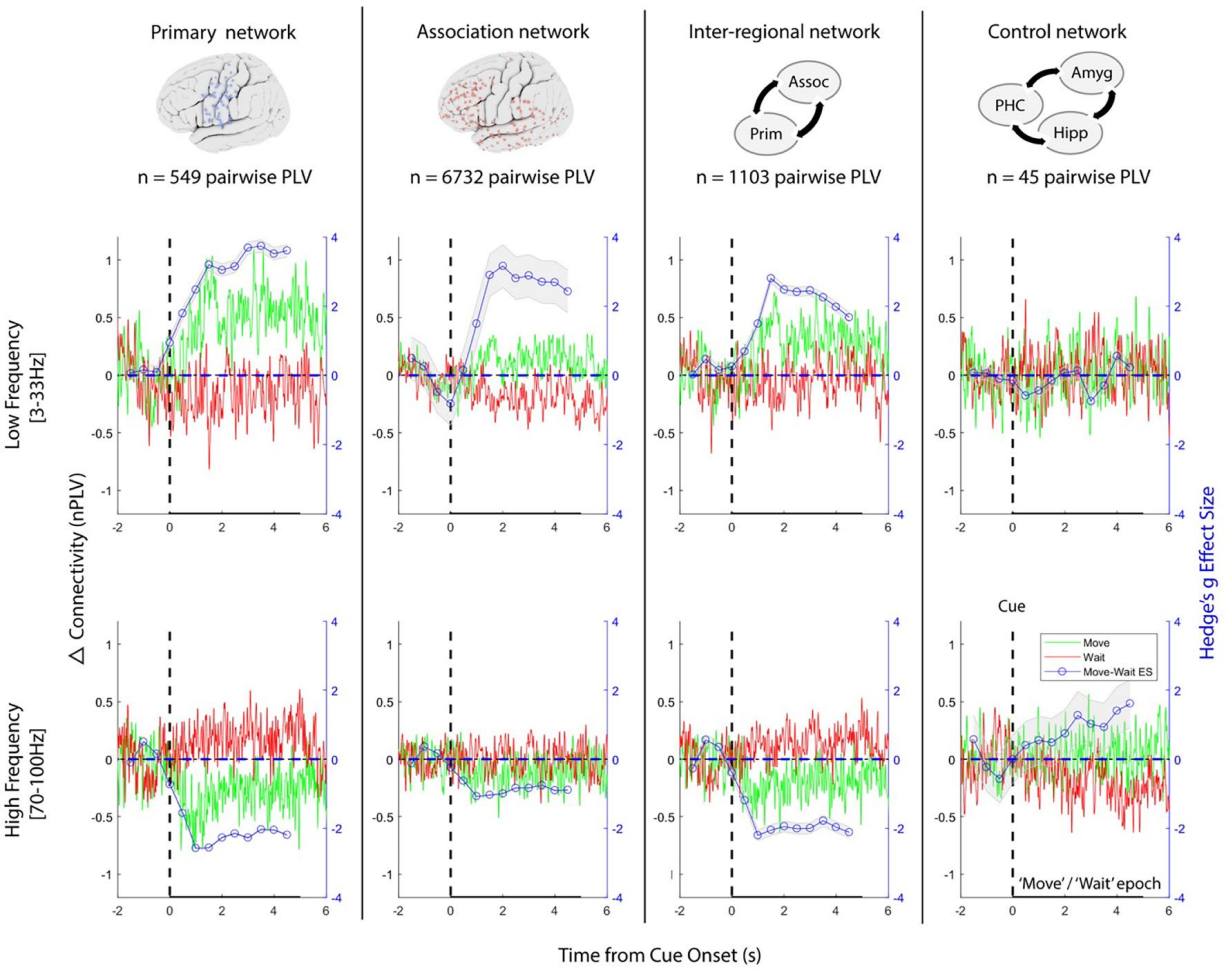
Anatomical distribution of pairwise connectivity profiles**.** Pairwise phase synchrony for low (top row) and high (bottom row) frequency activity averaged across all subjects and every pair of sites within the primary sensorimotor network (first column), within the association network (second column), across the sensorimotor and association networks (third column), and within the control limbic network (fourth column). Shown are the average change in PLV for both wait (red) and move (green) epochs, as well as the effect size of the difference between wait and move (blue).

We then wanted to understand if a similar phenomenon existed in the connectivity domain. Based on the strong anatomical breakdown of the spectral tilt clusters in our 5 subjects demonstrating task-modulation, we then focused on subject-specific anatomical connectivity within and between primary sensorimotor and association (other frontotemporoparietal) regions during movement versus rest epochs. We used all sites instead of just task-modulated sites to prevent a bias in the connectivity analysis towards a pre-specified spectral profile. As our connectivity metric, we assessed the statistical consistency of the phase of oscillatory activity between pairs of sites over time^13^. Following a previously described approach^13^, the pre-cue baseline normalized phase-locking value (PLV) was calculated for both low frequency (3–33 Hz) and high frequency (70–100 Hz) broadband oscillations for the duration of each cue-triggered ‘move’ and ‘wait’ epoch. For each subject, using the anatomical locations of each monopolar electrode contact, within-subject pairs were categorized as having both contacts within primary sensorimotor cortex (AA, 549 pairs, Table 1), both contacts within association regions (BB, 6732 pairs, Table 1), or one contact in primary cortex and the other in an association region (AB, 1103 pairs, Table 1). As a control, we generated a fourth, non-motor category comprised of connectivity between amygdala, hippocampus, and parahippocampal cortex (C, 45 pairs, Table 1). To quantify task-dependent, time-resolved connectivity changes between move versus wait epochs for each of these anatomical categories, the effect size of the difference in PLV across pairs and subjects was computed (Fig. 3).

**Table 1:** Anatomical breakdown of subject-specific electrode pairs.

Subject	Primary sites	Association sites	Mesial temporal sites	Primary pairs	Association pairs	Inter-regional pairs	Control pairs
1	4	57	6	6	814	16	15
2	31	68	2	175	927	190	1
3	30	67	8	163	1181	348	28
4	29	98	2	132	2361	509	1
5	18	73	0	73	1449	40	0
Total	112	363	18	549	6732	1103	4

**Figure 4:**
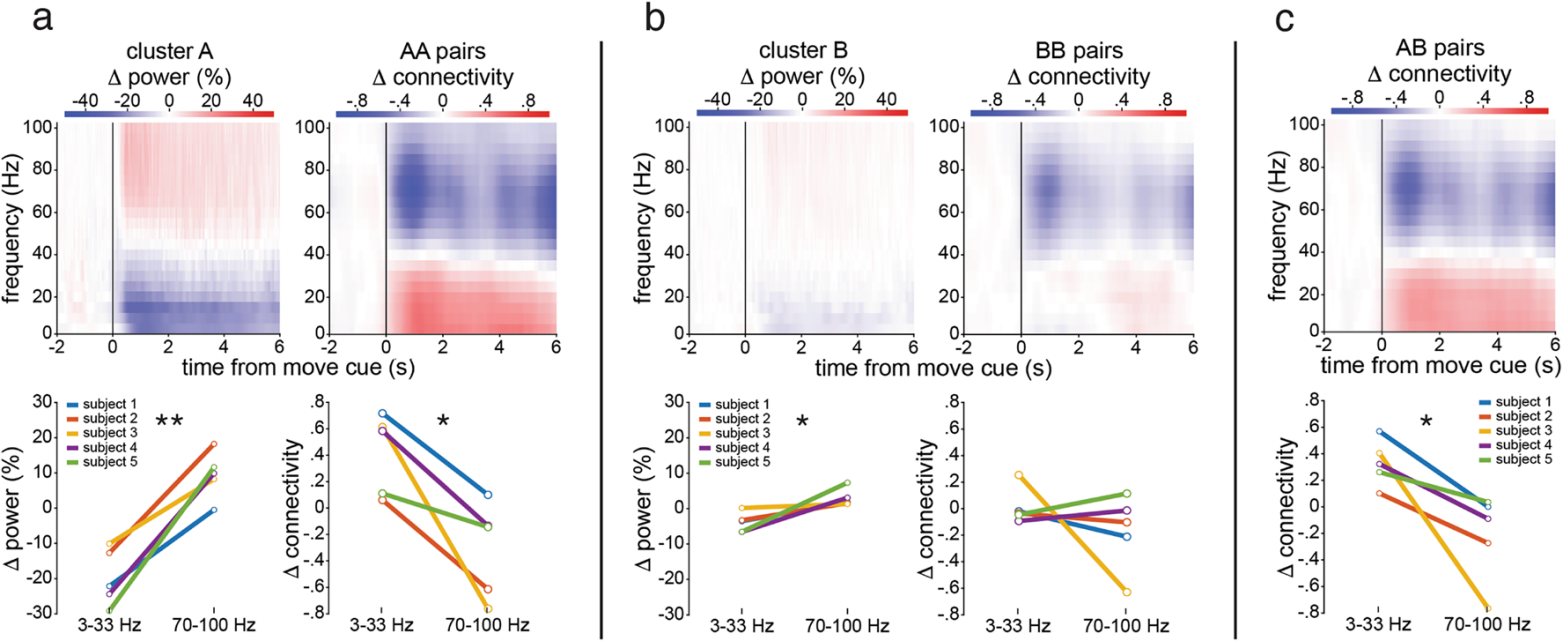
Induced spectral versus connectivity tilt in the sensorimotor network. (**a**, **b**, **c**) Change in power (left column) and connectivity (right column) during movement relative to 2 s before the move cue. Spectrograms of power and PLV (first row) were averaged across all subjects, electrodes/pairs, and trials after outlier removal. Average change in power and PLV averaged from the narrowband in low (3–33 Hz) and high (70–100 Hz) frequency bands shown for each subject individually during movement (1–5 s post cue, second row). Paired two-tailed t-tests were performed to assess statistical relationship for spectral and connectivity tilt. Asterisks represent *p*-values, ** = *p* < 0.01; * = *p* < 0.05. (**a**) Primary network (cluster A, AA interactions). (**b**) Associative network (cluster B, BB interactions). (**c**) Interactions between primary and associative pairs (AB interactions).

We found a frequency and task-dependent pattern of time-resolved functional connectivity that was graded based on the anatomical regions involved. In all but the control category, there was an increase in average low frequency PLV and a decrease in high frequency PLV during the movement epoch. The opposite was true during the wait epoch. The degree of modulation was dependent on anatomical region. Cue-triggered connectivity changes were most pronounced in primary sensorimotor pairs (category AA), followed by category AB, followed by category BB (Fig. 3). This pattern was not seen in the non-motor control network. To quantify this effect with higher frequency resolution and at the individual subject level, spectral and PLV analyses were broken down using non-overlapping 4 Hz windows, from 3 to 7 Hz (center frequency 5 Hz) to 98–102 Hz (center frequency 100 Hz). When utilizing this approach, the filter order for the PLV calculations were based on four cycles of the center frequency of each narrow band. This increased the variance in the distribution of the effect sizes particularly at lower frequencies, but overall demonstrated that theta frequency range as well has high beta range were the most prominent narrow bands driving the effect size difference in movement—wait epochs (Supp Fig. 1). Interestingly, theta range had a gradual ramp during the entire move epoch, whereas the high beta band was prominent immediately after movement onset then decayed (Supp Fig. 1a). This narrowband data was then used to derive the raw normalized changes in spectral power and PLV-based connectivity for the 5 s after movement onset normalized to the 2 s prior to movement onset. There was a clear inverse relationship in the average change in power versus PLV across time, frequency space, and anatomy (Fig. 4). The average change in LF versus HF power and connectivity in the primary sensorimotor area during movement was significant across subjects: t(4) = − 7.11, *p* = 0.0021 (power change) t(4) = 3.58, *p* = 0.0231 (connectivity change). The spectral tilt in task-modulated associative regions was also significant across subjects (t(4) = − 3.19, *p* = 0.0333) as was the connectivity tilt in all inter-regional interactions (t(r4) = 3.10, *p* = 0.0362). Statistically, spectral tilt showed a stronger effect due to the specific restriction of power-based clusters to task-modulated sites^23^. However, connectivity tilt was assessed across both task-modulated and non task-modulated sites based on pure anatomical localization to prevent a bias in the connectivity analysis towards a specific spectral profile. The transition point was approximately 35 Hz across both spectral and connectivity profiles.

Thus, as a near mirror image of the spectral tilt phenomenon, and without the pre-requisite of task-modulated spectral activation, there was an opposite “connectivity tilt”, seen most robustly in the primary sensorimotor region, followed by interactions between primary and associative regions, in which movement induced an increase in low-frequency phase synchrony and a decrease in high-frequency phase synchrony. This effect was equally present when using bipolar referencing (Supp Fig. 2). However, bipolar based referencing, particularly for pairwise PLV analyses in primary sensorimotor cortex, suffered from slightly less consistent individual patient data due to statistical restraints from the decrease in available anatomical pairs. Further, bipolar referencing may be suboptimal for connectivity analyses due to the subtraction of shared low frequency source information between electrode contacts, and false dampening of neural signal features from wide spatial distributions^26,27^.

## Discussion

Previous work has detailed the spectral activation pattern that correlates with motor cortical activation, known as spectral tilt^8^. This spectral tilt phenomenon, characterized by a decrease in low frequency power and concomitant increase in high frequency power, was first described in speech processing^28^, subsequently in memory encoding neurophysiology^29^, and is thought to represent local neuronal activity^29^. Using a data driven approach to separate functional clusters based on differential spectral activity profiles during move versus wait epochs, we confirmed the presence of the spectral tilt phenomenon in task-modulated nodes. Specifically, two clusters of activation profiles emerged, with Cluster A being a more robust spectral tilt profile arising heavily from primary sensorimotor cortices and putative human parietal-reach area^30^. Cluster B, which had a blunted spectral tilt pattern compared to Cluster A, significantly segregated into more associative regions, including frontal, temporal, and parietal distributed network nodes (Fig. 2).

However, instructed movement execution likely requires not only precise regional activation but also coordinated communication throughout the sensorimotor network. The precise frequency and anatomical dependency of time-resolved connectivity in the sensorimotor network during movement has not been fully elucidated. The change in phase-locking between two signals induced by a cue or stimulus (such as instructed movement onset) is a statistical measure of induced coordinated activity and argued to be a measure of functional connectivity^13,18,31^. Unlike previous methods of connectivity used in motor network electrocorticography (time-varying dynamic Bayesian networks)^19,20^, phase-based functional connectivity estimates provide a method to investigate the frequency-dependency of the coordinated network activity^13,32^. Interestingly, we found a frequency-specific, anatomically segregated connectivity pattern that differentiates movement (‘on-line’) versus wait (‘off-line’) periods. We found increased broadband low frequency (3–33 Hz) connectivity between activated sensorimotor network nodes, with strong contribution from the theta (3–8 Hz) range, similar to our previous work^21^ but also the high beta range (23–27 Hz, Supp Fig. 1), with a strong anatomical relationship driving the magnitude of change between movement versus rest periods (Fig. 3). Interestingly, the theta range demonstrated a gradual increase in effect size while the high beta range was most prominent immediately following the cue (Supp Fig. 1a). This is consistent with literature suggesting that beta rhythms are critical to synchronize sensorimotor neural populations for initiating normal movement^33,34^ and implicated in disorders of movement initiation such as Parkinson’s disease^35,36^. We further found that there was also a concomitant, robust decrease in high frequency broadband (70–100 Hz) connectivity in the same sensorimotor network nodes specific to movement (Fig. 3, Supp Fig. 1). Similar but in opposite direction as the spectral tilt phenomenon, we denote this relationship as the connectivity tilt (Fig. 4). Both the spectral and connectivity tilt phenomena characterize the ‘on-line’ sensorimotor network, and are strongly related to underlying anatomy. Similar to the spectral power domain, the magnitude of the connectivity tilt was most robust within primary sensorimotor cortical interactions (AA), and least robust in association region interactions (BB); while the magnitude of the connectivity tilt between association regions and primary cortex fell between the two (AB). Since PLV reflects the consistency of the phase difference between any two signals at every sample in time, there is a larger variance in the larger pairwise network (BB). However, this is accounted for in the error bars of the effect size (Fig. 3). Thus, even with the larger variance in effect size, the results very clearly still demonstrate the graded effect of the connectivity tilt from AA to AB to BB pairs. The phenomenon was specific to the task-relevant activated sensorimotor network, as the smaller but low variance mesial temporal network (control) did not exhibit this pattern. Of note, the number of pairwise PLV was vastly different between these different networks of interest. The error bars reflect these differences.

We found the transition point for the connectivity tilt effect to be approximately 35 Hz in the primary sensorimotor cortex (Fig. 4 and Supp Fig. 1). In frequencies below this transition point, movement was found to induce an increase in phase synchrony. Whereas in frequencies above the transition point, movement induced a decrease in phase synchrony. This combination of robustly opposite frequency-dependent spectral and connectivity tilt profiles characterizing the on-line sensorimotor network provides evidence that high frequency activity is largely asynchronous, stochastic behavior of activated neuronal populations. The asynchronous high frequency activity may then provide the opportunity for synchronizing cortical behavior in low frequencies to enable complex coordinated interactions.

Interestingly, similar, though not as well elucidated, findings have also been shown in presumptive memory circuits during a recall task^37^. The collective evidence may point towards a potential neurophysiological mechanism of how task-relevant brain regions activate and communicate to facilitate complex behavior. This mechanism involves both previously described spectral dynamics (spectral tilt) and our newly described phase dynamics (connectivity tilt) that may unite the spatially disparate nodes of the task-specific cortical network.

## Methods

### Subjects

Seven patients (1 female age 40, 6 males ages 24–52) undergoing intracranial clinical monitoring for intractable epilepsy were enrolled in this study. Electrodes were placed both subdurally and in deep temporal brain regions with placement determined by the clinical team to best localize epileptic focus. Data were collected at the Hospital of the University of Pennsylvania. Our research protocol was approved by the Institutional Review Board of the University of Pennsylvania Human Research Protections Program (Protocol #821,778, “The Epilepsy Research Collaborative for Patients Undergoing Surgical Intervention for Medically Intractable Epilepsy”). Informed consent was obtained from the participants or their guardians. All experiments were performed in accordance with relevant guidelines and regulations.

### Data collection

Electrodes consisted of 4-mm diameter platinum discs spaced 10 mm apart in a grid (8 × 8) or strip (1 × 4, 1 × 6, 1 × 8) arrangement embedded in silastic (Ad-Tech Medical Instrument Corporation, Racine, WI). Depth electrodes each had four contacts spaced 8 mm apart. There were a total of 493 contacts across 5 subjects who met inclusion criteria. There were a total of 18 depth electrode contacts in hippocampus, amygdala, or parahippocampal gyrus from 4 subjects. Data were recorded using a clinical Natus system (Natus Neurology, Middleton, WI), recording up to 128 channels of iEEG data at 512 Hz stored without and without reference to a common intracranial electrode chosen by the clinical team. To minimize heterogeneity, analyses used in the manuscript were performed on the raw non-re-referenced data, with post-processing and referencing performed as described in the spectral and connectivity analysis section. Analog pulses were sent from the behavioral presentation laptop to the clinical recording system to synchronize presented events with neural recordings.

### Task

A simple motor task was presented using a laptop during the epilepsy monitoring period, ranging from 2 to 14 days after implantation. Patients were directed to move either their right hand, left hand, or mouth and tongue in each trial with an on-screen instruction. Hand movements were demonstrated as opening and closing the hand and mouth and tongue movements were demonstrated as repetitive movement of the jaw and tongue without producing words or sound. Each trial consisted of three cues presented in sequence: a wait cue (“Wait for Instructions”), an instruct cue (e.g., “On ‘GO!’ please move your Right Hand”), and a move cue (“GO!”). Each screen was displayed for 5 s with twenty-four trials per session. The 5 s following each wait and move cue was considered the wait and move epoch, respectively (Fig. 1b). The wait epoch consisted of no movement behavior. The move epoch consisted of continuous designated movement behavior with cessation of movement after the 5 s epoch.

### Localization

A previously published open source automated platform^38^ was used to co-register post-operative computed tomography (CT) and magnetic resonance (MR) images to create a three-dimensional representation of each patient’s brain and electrode locations. Each patient’s preoperative MRI was registered with the Automated Anatomical Labeling (AAL) atlas using Advanced Normalization Tools (ANTS) to assign anatomical region label to each electrode and bipolar centroid as well as display electrode locations across subjects on the same standardized brain. The AAL merged atlas was used to group anatomical regions into primary sensorimotor cortex (S1/M1) or association regions (non-M1 frontal, temporal, or non-S1 parietal regions) for analysis of cluster distribution using a chi-squared test.

### Identification of task modulated sites

Similar to prior work, we first identified task-modulated recording sites to characterize the spectral profile of these activated regions^23^. Spectral power in the high gamma band (70–200 Hz), which we refer to as high frequency activity (HFA), was calculated during move and wait periods. Task-modulated sites were defined as those in which the mean difference in HFA between move and wait periods was significantly different from zero, using a two-tailed t-test with a false-discovery rate (FDR) corrected significance threshold of *p* = 0.05^23^. Five of the initial seven patients had task-modulated sites, which were included for subsequent analysis. Four of the these five also had depth electrodes with coverage of mesial temporal structures.

### Spectral analysis

For spectral analysis, a bipolar montage was used based on the geometric arrangement of contacts. Bipolar pairs were selected to include all immediately adjacent electrode pairs within 9.5–10.5 mm of each other^39^. Bipolar signals were obtained through raw subtraction of intracranial EEG signal and the recordings were denoised using a fourth-order butterworth notch filter at 60 + / − 2 Hz. The resulting data were treated as virtual electrodes (henceforth known as electrodes) originating from the midpoints between adjacent contacts^37^. Power was then calculated for each site in the bipolar montage by applying a Morlet wavelet convolution for frequencies 2–200 Hz on a log scale over the period 2 s prior to each cue onset and 6 s post-cue (+ / − 1-s buffer) using a 500-ms time window with 100-ms steps yielding 56 power calculations for 46 frequencies. Power was then z-score normalized for each subject using the mean and standard deviation calculated from 1000-ms windows randomly sampled at 60 + / − 10 s throughout the duration of the session^29,37^. Subsequently, extreme high amplitude artifacts were removed to ensure the data were robust to outliers by removing the top 8% of integrated spectral power data samples across all trials. On manual inspection, this was the optimal threshold to remove prominent high amplitude power artifacts. For direct comparison to the connectivity analysis, the bipolar data for each 8 s window (− 2 to 6) around the cue was re-normalized to the 2 s prior to the cue, and assessed over the 6 s post cue. This was additionally compared to a monopolar montage with common average re-referencing. In this analysis, power was averaged in broadband low (3–33 Hz) and high (70–100 Hz) frequencies for the 1–5 s post movement, as well as in non-overlapping 4-Hz narrowbands centered every 5 Hz from 5 Hz [3–7 Hz] to 100 Hz [98–102 Hz].

### Connectivity analysis

Monopolar montage with common average referencing was used for pairwise phase synchrony analysis using phase-locking value^13^. Bipolar based referencing (Supplemental Fig. 2) was also compared. Bipolar referencing is particularly tailored for identifying isolated focal high frequency signals, and can subtract true shared source information, especially low frequency components that derive from a larger geometric area, between neighboring contacts^26,27^. Based on a consensus guidelines and good practices, this makes bipolar referencing susceptible to dampening neural signal features from wide spatial distributions^27^. Further, bpolar referencing decreases the number of pairwise interactions that can be analyzed. Thus monopolar referencing with common average referencing was preferred to minimize bias in long-range low frequency interactions and maximize the number of contacts available for pairwise statistical analyses within-subject. Monopolar recordings were denoised using a fourth-order butterworth notch filter at 60 + / − 2 Hz. Based on anatomical localization of each monopolar contact, the within-subject pairs were assigned as primary-primary (“primary network”, n = 549 pairs across subjects), association-association (“association network”, n = 6732 pairs), and primary-association (“inter-regional network”, n = 1103 pairs). As a comparison non-motor network for these analyses, a mesial temporal group was created using any within-subject pairs of contacts in hippocampus, amygdala, or parahippocampal gyrus (“control network”, n = 45 pairs) (Table 1). Low (3–33 Hz) and high (70–100 Hz) frequency broadband signals were extracted from the raw time series data of each electrode using finite-impulse response filters of order 250 and 50, respectively. Additionally, the data was also broken down into 4-Hz frequency bands, centered every 5 Hz from 5 to 100 Hz, with finite-impulse response filter orders set to equal 4 cycles of the center frequency of each band at 512 Hz sampling rate. In both broadband and narrowband analyses, these signals were Hilbert transformed to obtain instantaneous phase data. The phase-locking value (PLV)^13^ was then calculated across time between each monopolar electrode contact (henceforth known as contact) pair, regardless of geometric distance as this does not affect phase-related synchronization measurement^13,40,41^. Task-related PLV changes for each contact pair were assessed in cue-aligned epochs (two seconds before to six seconds after each move and wait cue) averaged across trials. Again to ensure robustness of outliers, the top 5% of absolute PLV values were removed across trials which corresponded visually to an optimal removal of clear outlier values. To combine these data across all pairs and subjects, the PLV values were z-score normalized using the mean and standard deviation calculated from the 2 s prior to the cue. To quantify how connectivity changes differed throughout the move and wait epochs, the effect size was calculated using Hedges’ g^42,43^ in a sliding window (1-s width, 0.5-s step). Hedge’s g effect size was also averaged for each 4 Hz frequency band across the 1–5 s post movement or wait cue. For the PLV spectrogram plots, the PLV values were smoothed with a sliding time–frequency window of 500 ms and 8 Hz. For direct comparison to the power analysis, PLV was also then averaged in broadband low (3–33 Hz) and high (70–100 Hz) frequencies from 1 to 5 s after movement onset.

### Other statistical analyses

K-means clustering was used to segregate task-modulated patterns of spectral profiles during movement into Cluster A and Cluster B using the Calinski–Harabasz criterion^24,25,44,45^. Paired two-tailed t-tests were used to assess the average change in low frequency versus high frequency power and connectivity during movement across subjects.

### Supplementary Information

Supplementary Figure 1.

## Data Availability

All data needed to evaluate the conclusions in the paper are available in the main text. The dataset is available from the corresponding authors upon request.
