# Supplementary material for: Dichotomous frequency-dependent phase synchrony in the sensorimotor network characterizes simplistic movement
Source: Sci Rep. 2024 May 24;14:11933. doi: 10.1038/s41598-024-62848-9 (PMC11126677; doi:10.1038/s41598-024-62848-9)
Supplement: Supplementary file 1 — Supplementary Figure 1. [file 41598_2024_62848_MOESM1_ESM.docx]

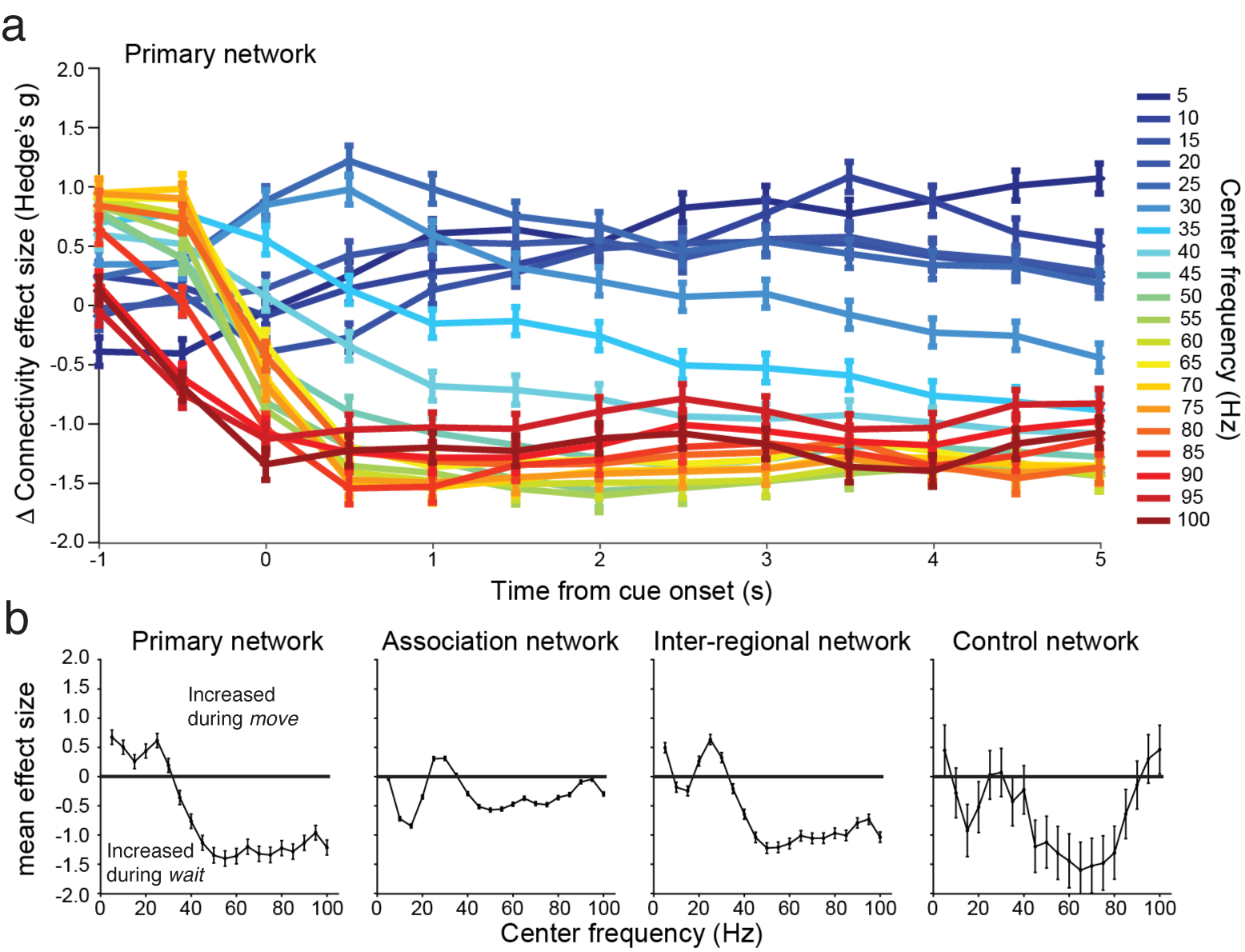


**Supplemental Figure 1. Move - Wait effect size across frequencies.** a) Effect size (Hedge’s g) of primary network (AA) interactions during movement compared to wait periods from 1 second before to 5 seconds after cue. There is a gradient change in in effect size from low frequencies (blue) to high frequencies (red), where low frequencies have an increase in effect size from 0-5s, while high frequencies have a decrease effect size. There is a potential bimodal peak in the low frequencies, where theta/alpha values slowly ramp during the entire epoch, while high beta values are most critical immediately at movement onset. The transition point in this plot is around 35Hz, which is closest to 0. b) Averaged effect size during the 1-5 seconds post-cue, now for all of the network interactions being analyzed. Positive effect size implies the average effect was higher during movement in that frequency band. Negative effect size implies the average wffect was higher during wait epochs in that frequency band. There is a bimodal peak in low frequency where theta and high beta are the highest, while gamma frequencies are more uniform. This is most robust in primary-primary and primary-associative interactions. Control network interactions are more prominent during wait period across frequencies.


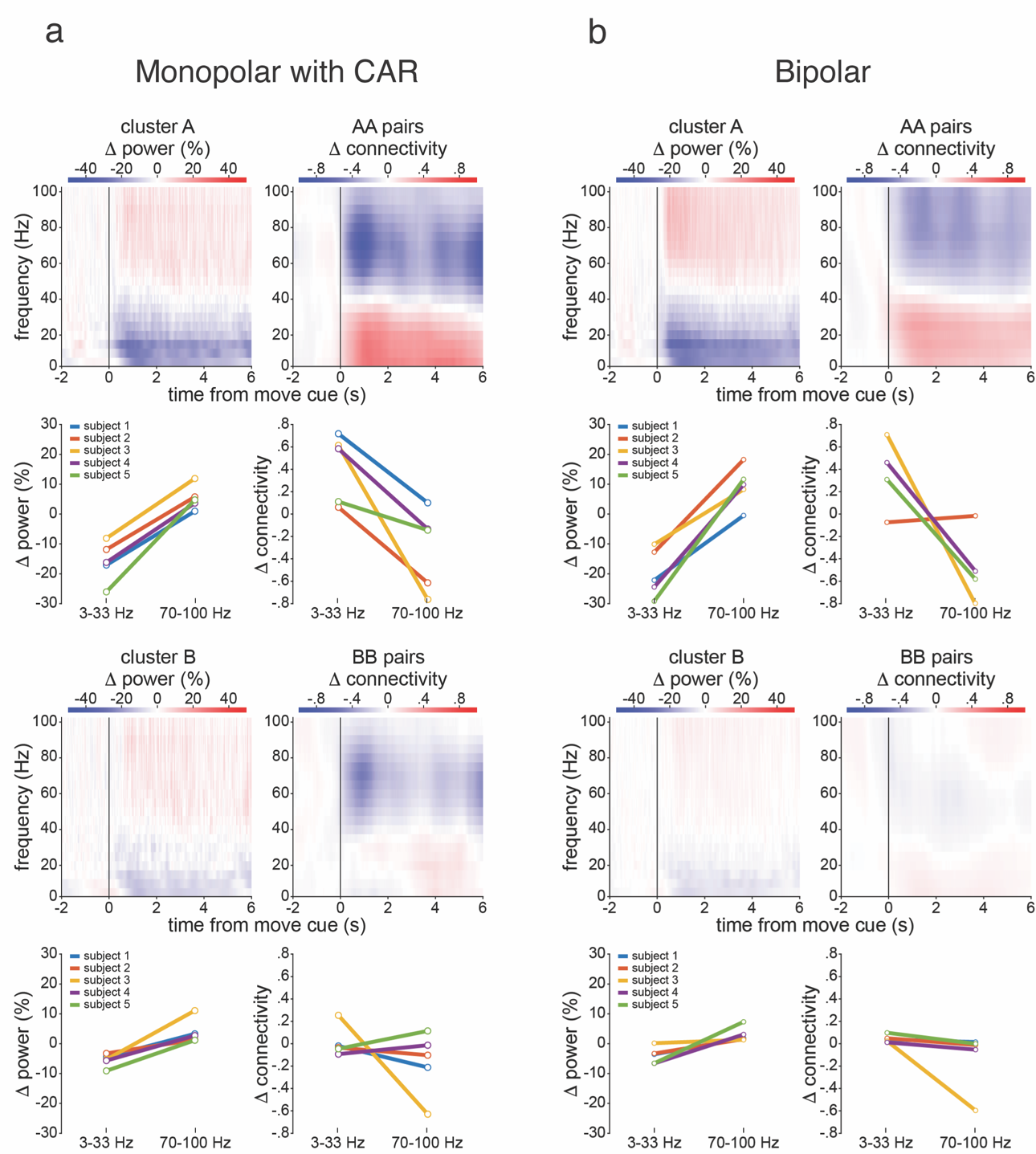


**Supplemental Figure 2. Monopolar with CAR versus Bipolar re-referencing for induced spectral and connectivity tilt.** a,b) Left column represents changes in power, while right column represents changes in connectivity. Top half of plot are primary network cluster and cortical interactions, while bottom half represents associative network cluster and cortical interactions. a) Monopolar CAR and b) Bipolar. When comparing the two methods, average spectrograms across the 4 conditions are similar. In both, the AA connectivity profiles have a slightly more robust differentiation compared to cluster A power. Cluster B power and BB interactions are slightly more robust in monopolar compared to bipolar. The primary network connectivity tilt has a robust response similar to monopolar, but the effect is lost completely in one subject. Associative network power and connectivity are similar, though connectivity has slightly more variability in high frequency PLV with monopolar CAR.
